# Oil-Dispersible Green-Emitting Carbon Dots: New Insights on a Facile and Efficient Synthesis

**DOI:** 10.3390/ma13173716

**Published:** 2020-08-22

**Authors:** Gianluca Minervini, Annamaria Panniello, Elisabetta Fanizza, Angela Agostiano, Maria Lucia Curri, Marinella Striccoli

**Affiliations:** 1 Chemistry Department, University of Bari “Aldo Moro”, Via Orabona 4, 70126 Bari, Italy; g.minervini8@studenti.uniba.it (G.M.); elisabetta.fanizza@uniba.it (E.F.); angela.agostiano@uniba.it (A.A.); 2 Italian National Interuniversity Consortium of Materials Science and Technology (INSTM) Bari Unit, c/o Chemistry Department, University of Bari “Aldo Moro”, Via Orabona 4, 70126 Bari, Italy; 3 CNR-IPCF-Bari Division, c/o Chemistry Department, University of Bari “Aldo Moro”, Via Orabona 4, 70126 Bari, Italy; m.striccoli@ba.ipcf.cnr.it

**Keywords:** carbon dots, one-pot synthesis, green synthesis, luminescent nanostructures

## Abstract

Carbon dots (CDs) have been progressively attracting interest as novel environmentally friendly and cost-effective luminescent nanoparticles, for implementation in light-emitting devices, solar cells, photocatalytic devices and biosensors. Here, starting from a cost-effective bottom-up synthetic approach, based on a suitable amphiphilic molecule as carbon precursor, namely cetylpyridinium chloride (CPC), green-emitting CDs have been prepared at room temperature, upon treatment of CPC with concentrated NaOH solutions. The investigated method allows the obtaining, in one-pot, of both water-dispersible (W-CDs) and oil-dispersible green-emitting CDs (O-CDs). The study provides original insights into the chemical reactions involved in the process of the carbonization of CPC, proposing a reliable mechanism for the formation of the O-CDs in an aqueous system. The ability to discriminate the contribution of different species, including molecular fluorophores, allows one to properly single out the O-CDs emission. In addition, a mild heating of the reaction mixture, at 70 °C, has demonstrated the ability to dramatically decrease the very long reaction time (i.e. from tens of hours to days) at room temperature, allowing us to synthesize O-CDs in a few tens of minutes while preserving their morphological and optical properties.

## 1. Introduction

Carbon dots (CDs) are novel photoluminescent nanoparticles, increasingly emerging as active materials in many application areas, such as optoelectronics, solar cells, catalysis, sensors and bioimaging [1,2,3,4,5,6]. Unlike the inorganic semiconductor quantum dots, the structure of the CDs is entirely carbon-based, hence they are heavy-metal free, and combine good photostability and high quantum yields [2,3] with improved biocompatibility and low associated environmental risks [4,5,7]. Moreover, a key advantage of the CDs is the option of preparing them from green and low-cost sources [8,9], via rather simple bottom-up procedures. Common CDs synthetic approaches rely on hydrothermal-, solvothermal- or microwave-based methods, starting from a carbon precursor (e.g., citric acid, carbohydrates or other organic compounds, also present in natural extracts of fruits, plants, leaves), that is treated at an elevated temperature and pressure in an autoclave, or exposed to microwaves [8,10,11,12,13,14]. Typically, alongside the main carbon precursor, other components are added to the reaction mixtures to enhance surface passivation of the forming CDs, or to introduce heteroatoms in the carbogenic structure, in order to improve their photoluminescence (PL) [15,16]. In particular, nitrogen is by far the most common of such “dopant” heteroatoms, as, in many cases, the presence of amine, amidic, pyridinic or pyrrolic groups in the CD core has been demonstrated to significantly enhance their photoluminescence quantum yield (PLQY) [15,17,18,19,20,21,22]. More recently, a cationic surfactant, namely cetylpyridinium chloride (CPC) (inset Figure S1), was proposed as a novel precursor for the synthesis of CDs [23,24,25]. Indeed, the pyridinium polar head-group of the CPC is a nitrogen-containing heteroaromatic system, thus, in principle, very suitable for the formation of extended conjugated aromatic structures in the CD core. On the other hand, the alkyl chains in the compound are expected to play a key role in the stabilization and passivation of the CD’s surface. Therefore, CPC can be regarded as an all-in-one precursor candidate for the preparation of surface passivated, and inherently nitrogen featuring, CDs. This amphiphilic molecule was firstly proposed by Zbořil et al. for the preparation of CDs by means of a hydrothermal method [23]. However, later, Zheng et al. [24] demonstrated the possibility of using the CPC to synthesize green-emitting CDs in a very easy and cost-effective method that circumvented the need for energy-consuming hydrothermal treatments. Indeed, CDs were obtained simply by adding concentrated NaOH solutions to CPC aqueous solutions without supplying any further external energy. In particular, the approach proposed by Zheng et al. allows the synthesis of either water-dispersible (W-CDs) or oil-dispersible (O-CDs), depending on the reaction time and on the concentration of the added NaOH solution (C_NaOH_) [24]. Specifically, after a short reaction time (≈1 h) from the NaOH addition, W-CDs are obtained, while, as the reaction proceeds, the pristine W-CDs are increasingly converted into O-CDs. O-CDs can be thus isolated after long reaction time, ranging from 18 h to several days, as a function of the C_NaOH_. Typically, the addition of a more concentrated NaOH solution enables a faster formation of O-CDs. Therefore W-CDs, which show biocompatibility and low cytotoxicity, can be obtained by quenching the reaction early enough to prevent their conversion into O-CDs. Nevertheless, such W-CDs display a rather low PLQY (<10%). Conversely, O-CDs present higher PLQY and can be dispersed in various common organic solvents, which makes them particularly suitable for their integration into fabrication processes of optoelectronic devices and sensors [24,25]. However, the conversion of the W-CDs into O-CDs is, in general, a time-consuming procedure when performed at room temperature (RT). Therefore, such a synthetic approach to obtaining O-CDs would greatly benefit, in terms of feasibility and convenience, from a reduction in the reaction time. In order to accomplish such a goal, it is essential to gain an understanding of the chemistry involved in the CPC carbonization that leads to the formation of CDs, already in ambient conditions, unlike other common carbon precursors. The formation of W-CDs and O-CDs from the reaction of CPC with NaOH, which can be explained by considering the specific chemical reactivity of CPC in the presence of OH^−^ ions [23,24,25], still lacks a comprehensive and detailed description. In fact, a fundamental understanding of the synthetic processes is essential in order to direct the synthesis to the achievement of CDs with well-defined properties that may, finally, address technological applications. Furthermore, it has been widely shown, in the hydrothermal or microwave-assisted syntheses, that fluorescent molecules can be originated alongside the CDs in the reactions, and it is commonly accepted that such species significantly contribute, becoming even predominant, to the overall emission of the CDs [26,27,28,29,30,31]. Therefore, the identification of such emitting species and their comprehensive spectroscopic characterization is fundamental in order to distinguish the CDs’ emission contribution. In turn, the ability to isolate the CDs and to properly assess their PL properties is the primary requirement in evaluating the origin of their emission. Indeed, the foundation of the CDs’ fluorescence is still an open question, being widely debated in the scientific community [32,33,34,35,36,37,38]. While for semiconductor quantum dots the emission is an intrinsic property, being mainly due to the quantum confinement effect, in the case of CDs it has been demonstrated that the surface chemistry plays a decisive role on their spectroscopic behavior, besides the contribution of the intrinsic emission from the carbogenic core. Here, we investigate the chemical processes, starting from CPC as a convenient precursor for an easily implementable and cost effective and sustainable procedure, that lead to the formation of the O-CDs, paying specific attention to the reactions taking place in the solution upon NaOH addition. We demonstrate the formation of blue-emitting molecular fluorophores alongside the CDs that behave as intermediates in the synthesis and contribute to their overall emission. These new insights into the chemistry occurring in the system allow us to depict an alternative reaction mechanism that relies on the consumption of the blue-emitting intermediates to generate the green-emitting O-CDs. The described chemical reactions and reaction mechanism also illustrate the reasons why the CPC is so effective in the realization of the CDs, as it is based on the peculiar reactivity of its pyridinium polar head and long alkyl chain.

Finally, a mild heating of the reaction mixture is found to be able to greatly reduce the reaction time, yielding O-CDs with unmodified green emissions already within 20 min, instead of the long reaction time necessary for the room temperature synthesis, thus further verifying the proposed mechanism, and proving that the spectroscopic features are related to the degree of carbonization achieved during the synthesis, irrespective of the experimental parameters.

## 2. Materials and Methods 

### 2.1. Materials

Cetylpyridinium chloride (CPC), sodium hydroxide (NaOH) and hydrochloric acid (HCl) were purchased from Merck (Sigma-Aldrich, Milan, Italy) and dissolved in MilliQ water (Sigma Aldrich, Milan, Italy). All solvents used for separation and purification procedures and for spectroscopic characterization were of analytical grade; chloroform (CHCl_3_), methanol (MeOH), acetone (Act), n-hexane (n-Hex), tetrahydrofuran (THF), acetonitrile (AcN) and N,N-dimethylformamide (DMF) were purchased from Merck (Sigma-Aldrich, Milan, Italy), whereas dichloromethane (CH_2_Cl_2_) was purchased from Fluka (Honeywell, Milan, Italy). All listed chemicals were used as received, without any further purification.

### 2.2. Synthesis of CDs

Firstly, the CDs were synthesized according to the method reported by Zheng et al. [24], consisting of a simple reaction of CPC with NaOH at RT. The reaction was performed in common glass vials in air atmosphere, by adding NaOH aqueous solution to CPC aqueous solution. The final C_NaOH_ was varied in the range of 90–360 mM, whereas the final concentration of CPC was kept fixed at 15 mM. This concentration is higher than the critical micelle concentration of CPC, generally estimated to be of 1 × 10^−4^–1 × 10^−3^ mol∙kg^−1^ in the absence of other ionic species [39,40,41]. This ensures the presence of CPC (in the form of micelles) in the bulk of the solution during the synthesis. The synthesis of CDs was also performed at a slightly increased temperature (40 and 70 °C). In this case, the CPC aqueous solution was initially heated at the selected temperature in a thermostatic bath, and subsequently, the NaOH solution was added. The temperature was kept constant at the same value during the reaction. The reaction was quenched at the desired time by adjusting the pH to neutral (pH = 7), through addition of HCl. A more detailed description of the purification protocols is reported in the supplementary materials.

### 2.3. Post-Synthetic Purification

In general, the reaction product results in a mixture of W-CDs and O-CDs [24]. To separate the two different types of the obtained CDs, a post-synthetic procedure was required, consisting of the addition of a fixed volume of CHCl_3_ or CH_2_Cl_2_ and centrifuging until obtaining a complete separation of the water and oil phases. Subsequently, the W-CDs were purified by dialysis, and the O-CDs were dried under nitrogen flow and then washed by using different organic solvents (MeOH, Act, n-Hex, THF, AcN and DMF). More details are reported in the supplementary materials.

### 2.4. Transmission Electron Microscopy Characterization

Transmission electron microscopy (TEM) analysis was carried out using a JEOL JEM1011 microscope (JEOL, Akishima, Tokyo, Japan)., equipped with a LaB_6_, electron source operating at an accelerating voltage of 100 kV and using carbon-coated copper grids. The images were acquired using an Olympus Quemesa CCD camera (Olympus, Münster Germany). The statistical analysis of the samples was performed using the ImageJ freeware image analysis program on a relevant CDs population (about 400 counts) to evaluate the nanoparticle average size and the percentage relative standard deviation (σ%) for estimating the nanoparticle size distribution.

### 2.5. Spectroscopic Characterization

UV-Vis absorption spectra were recorded with a Cary 5000 (Agilent Technologies, Inc., Santa Clara, CA, USA) UV/Vis/NIR spectrophotometer. PL emission spectra were acquired using a Fluorolog 3 spectrofluorometer (HORIBA Jobin-Yvon GmbH, Bensheim, Germany), equipped with a 450 W Xe lamp as excitation light source, and double-grating excitation and emission monochromators. Absolute PLQY measurements were performed using a “Quanta-phi” integration sphere coated with Spectralon^®^ (HORIBA Jobin Yvon GmbH, Bensheim, Germany) (reflectance ≥95% in the range 250–2500 nm).

## 3. Results and Discussion

### 3.1. O-CD Synthesis

**Figure 1:**
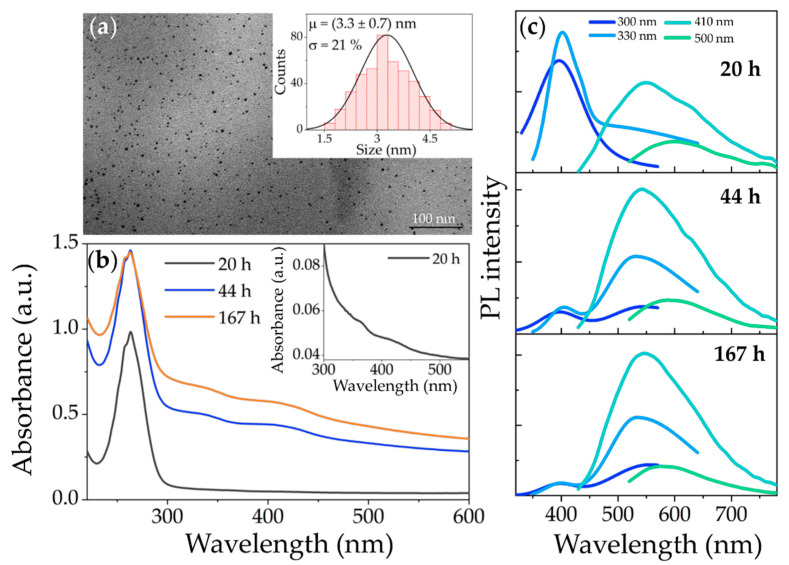
(**a**) TEM micrograph and size distribution (inset) of O-CDs synthesized at RT. The scale bar is 100 nm; (**b**) UV-Vis absorption spectra of a representative sample (CPC aqueous solution after addition of 90 mM NaOH) at increasing reaction times. For sake of clarity, the region above 300 nm of the spectrum recorded at 20 h is shown with a higher magnification in the inset; (**c**) PL spectra at different excitation wavelengths recorded for the samples in (**b**).

CDs have been synthesized using a simple and user-friendly approach based on the carbonization of CPC (15 mM aqueous solution) upon NaOH addition [24,25]. The process leading to the O-CDs at RT, according to the method reported in [24], has been investigated as a function of C_NaOH_. After 167 h (≈7 d), spheroidal shaped O-CDs were obtained, with an average size of 3.3 nm (σ = 21%), irrespective of the used C_NaOH_ (Figure 1a).

After the NaOH addition, the reaction was monitored over time by recording UV-Vis and PL spectra of the reaction mixture, in order to investigate luminescent species originating and/or consumed during the reaction.

The UV-Vis absorption spectra (Figure 1b) clearly display a peak at around 260 nm, and two faint bands at 360 and 420 nm. In the literature, for CDs synthesized with diverse approaches, the signal below 300 nm in the absorption spectrum is commonly ascribed to the characteristic π-π* transitions of the carbogenic cores of the CDs, due to the conjugated sp^2^-carbon domains [27,29,42,43,44,45]. Moreover, in such a spectral range, the π-π* absorption transitions of the polyheteroaromatic structures derived from pyridinium units of the CPC head-group can also occur. Therefore, the absorption signal at 260 nm can additionally be indicative of some unreacted carbon precursor [46]. Such an attribution is confirmed by the UV-Vis absorption spectrum of a bare CPC solution (Figure S1) that displays the characteristic absorption band in the UV range, while no other absorption feature can be detected in the visible portion of the spectrum.

The two absorption bands at 360 and 420 nm become evident only 20 h after the starting of the reaction at RT (Figure 1b), and can be safely ascribed to the CDs formed during the carbonization of the CPC upon the addition of NaOH, as demonstrated in [24]. In particular, such bands are common to both the W-CDs and the O-CDs in the reaction mixture at the investigated time. In fact, after the separation, both the types of CDs show similar absorption bands in the visible range, distinct from the chemical groups or structures forming the CDs, in agreement with [24]. However, the possibility of safely inferring the exact origin of the electronic transitions giving rise to these bands is not trivial: similar absorption bands above 300 nm are often related to the n-π^*^ transitions of the surface-related chemical groups of the CDs that form a system of multiple discrete energy states [29,43,44,47,48,49]. However, other reports [24,25,50,51] ascribe them to the π-π* and n-π* transitions, respectively, of the characteristic interconnected heteroaromatic units of the CDs. It is also worth noting that all the absorption spectra show a characteristic scattering background that decreases monotonically at increased wavelengths. Such a scattering contribution, growing with the reaction time, as also evident from the turbidity of the mixture increasing as the reaction proceeds, can be accounted for by the formation of an O-CDs-containing insoluble phase dispersed in the aqueous medium.

The PL spectra of the reaction mixture have been recorded in the range of 300–500 nm at different times after the NaOH addition, as a function of the excitation wavelength (λ_exc_), in order to assess a possible excitation dependence, which is known as a peculiar feature of the CDs [26,28,29,30,42,44]. The spectra (Figure 1c) exhibit two emission signals, namely i) a band positioned at 400 nm, irrespective of the λ_exc_, that weakens in intensity at increasing reaction times, and ii) a broader excitation dependent band, peaking at 550 nm for λ_exc_ in the range 300–410 nm, which here red-shifts up to 590 nm for λ_exc_ = 500 nm. Such an excitation-dependent band is typically observed in the spectra of CDs obtained from reactions using CPC as a precursor [23,24,25]. In fact, the PL spectra of a bare CPC solution (Figure S1b) do not show any emission bands in the spectral range of 300–700 nm, thus allowing us to safely rule out any possible attribution to the unreacted precursor. Interestingly, the excitation-independent PL band at 400 nm has been found to significantly contribute to the spectra mainly at short reaction times, as evident in the spectrum recorded at 20 h, whereas its intensity decreases at longer times (Figure 1c). In the literature, the presence of a PL band below 500 nm, though excitation-dependent, has been reported for CDs synthesized using CPC and NaOH at elevated temperature and pressure using a hydrothermal method [23], these being attributed to core-state transitions of the resulting CDs. However, an emission band in the blue region has not been measured so far for CDs synthesized at RT and under atmospheric pressure [24,25]. 

Here, the observed excitation wavelength-independence of the emission band at 400 nm, and its drop in intensity with prolonged reaction time, suggests that such a PL contribution originates from intermediate species forming in the early reaction step, which are then consumed to produce the green-emitting CDs as the reaction proceeds. In particular, it is reasonable to assume that the blue emission is due to fluorescent species originating during the CPC carbonization, such as 2-pyridone derivatives of CPC. Indeed, 2-pyridones are a prominent class of fluorophores, well known, inter alia, as dyes, pigments or fluorescent bio-markers [52,53], which have been reported to form upon oxidation or disproportionation reactions, resulting from the treatment of pyridinium salts with NaOH under mild conditions [54]. In general, the formation, alongside the CDs, of blue-emitting molecular fluorophores has been largely demonstrated in popular CDs synthetic approaches based on the carbonization of citric acid in the presence of di- or triamines [26,27,28,29,30,31]. In particular, blue-emitting 2-pyridone fluorophores, such as citrazinic acid or imidazo[1,2-α]pyridine-7-carboxylic acid, 1,2,3,5-tetrahydro-5-oxo- (IPCA), have been identified and isolated [26,27]. The 2-pyridone molecular fluorescent species are, then, expected to form here as well, as reaction intermediates in the investigated approach based on the carbonization of CPC.

Therefore, the PL spectra of the reaction mixture in Figure 1c can be explained considering the contributions of three different emitting species: the W-CDs, the O-CDs and the 2-pyridone intermediates. Particularly interesting is the investigation of the emission properties of the O-CDs, as they present a PLQY higher than that characterizing the W-CDs [24,25]. In addition, their possible dispersion in organic solvents allows a remarkable processability, which makes them versatile in various areas of applications. It is therefore necessary to single out the O-CDs’ specific emission contribution. In order to separate the different contributions, an O-CDs extraction procedure from the aqueous reaction environment also containing W-CDs was performed. For this purpose, a water immiscible solvent (e.g., CHCl_3_ or CH_2_Cl_2_) was added to the system, which was then centrifuged to separate the aqueous and oil phases, finally isolating the O-CDs-containing oil phase. Then, a series of solvents, such as methanol, acetone, n-hexane, tetrahydrofuran, acetonitrile and N,N-dimethylformamide, was tested to treat the extracted O-CDs in order to dissolve the 2-pyridone derivatives, while leaving undispersed the O-CDs, which could thus be isolated in the precipitate by centrifugation.

Interestingly, the O-CDs have been shown to be, to some extent, dispersible in all the tested solvents. Such behavior is compatible with the characteristic heterogeneity in the surface composition of the CDs, where both polar and apolar groups (e.g., ─COOH, ─C═O, ─OH, ─C═N or CPC residual alkyl chains [24,25]) make the O-CDs dispersible in a variety of solvents with different polarities.

**Figure 2:**
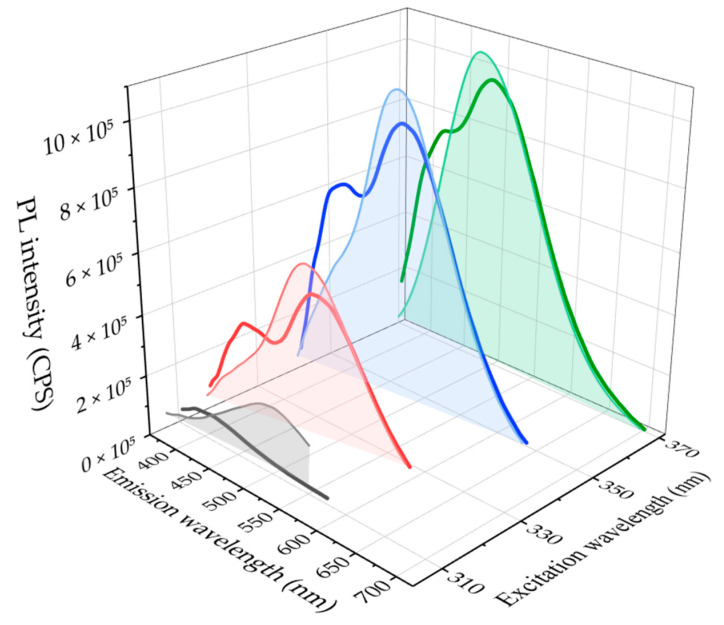
Comparison of the PL spectra of a purified O-CD sample re-dispersed in CHCl_3_ (filled area) and the respective supernatant (acetone based) after the purification procedure (thick lines) recorded at different excitation wavelengths.

Figure 2 shows a comparison between the PL spectra of a purified O-CD sample, re-dispersed in CHCl_3_, and the spectrum of the relative supernatant (non-solvent based) obtained after centrifugation, recorded at different λ_exc_. The PL spectra of both the supernatant (thick lines) and the CHCl_3_ dispersion of purified O-CDs (filled area) exhibit two contributions in the blue and in the green regions, respectively, at around 400 nm due to the 2-pyridone molecular intermediates, and at around 520 nm (λ_exc_ = 310–370 nm), ascribed to the O-CDs.

However, the intensity of the blue emission is considerably weaker than the green one for the re-dispersed purified precipitate at each investigated λ_exc_, while the supernatant spectra exhibit a much more intense blue emission, due to the 2-pyridone fluorophores. Such an evidence confirms that the solvents used in the purification step are able to dissolve and remove most of the blue-emitting species. In particular, deconvolutions of the PL spectra (Figure S2) demonstrate that the overall area associated with the emission of the residual blue-emitting intermediates in purified O-CDs is less than the 10% of the overall area of the PL spectrum, at all the investigated λ_exc_ values. The absolute QY of the purified O-CDs measured at 410 nm is 16 ± 1%, in agreement with the work of Zheng et al. [24], while the value for the W-CDs is limited to a few percent. The spectral deconvolution of the PL band for O-CDs is also used to assess a reliable value of the QY of the green emission of the O-CDs only, estimating the contribution of the emission of the blue fluorophores that results in the experimental error on the measure. The implemented purification procedure is effective in removing most of the blue-emitting species from the samples, allowing us to isolate the O-CDs and to highlight their green-emitting contribution to the overall fluorescence. 

#### Chemical Insights on the CPC Carbonization Process

**Scheme 1:**
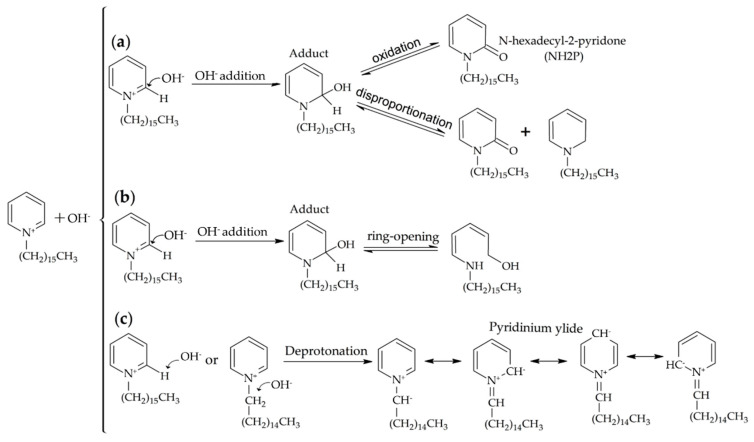
Chemical reactions taking place upon addition of NaOH to CPC solution under mild conditions. (**a**) Oxidation and disproportionation reactions leading to the formation of the blue-emitting 2-pyridones; (**b**) ring-opening reactions; (**c**) deprotonation reactions yielding a resonance-stabilized ylide.

The chemistry taking place when NaOH is added to the CPC solution deserves particular attention in order to elucidate the mechanism that, starting from the amphiphilic CPC molecules, leads to the formation, first, of the blue-emitting intermediates, and finally of the green-emitting O-CDs. A comprehensive picture of the reactivity of the CPC molecules in the presence of OH^−^ under mild conditions is shown in Scheme 1. According to path (a), the addition of the OH^−^ at positions 2 or 4 of the CPC aromatic ring, favored by the positive charge present on the nitrogen atom, leads to the formation of an adduct [54]. Such an adduct can subsequently undergo oxidation (or disproportionation), to give a 2-pyridone head-grouped amphiphilic molecule (N-heaxdecyl-2-pyridone, NH2P). Alternatively, the adduct can evolve by ring-opening reactions to give-double bond-containing products (path (b)). Finally, according to path (c), deprotonation reactions occur by effect of the hydroxide ions. In particular, α-carbon either in the pyridinium aromatic ring or in the alkyl chain can deprotonate, yielding a resonance-stabilized pyridinium ylide that features a negatively charged carbon atom bound to a positively charged nitrogen atom. Such deprotonation reactions can be plausibly assumed to be the origin of the CPC carbonization process leading to the CDs [23].

**Scheme 2:**
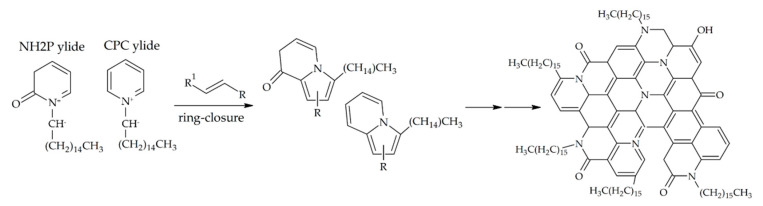
Proposed reaction mechanism based on reactions of deprotonated CPC or NH2P molecules with dipolarophile ring-opening products, yielding extended polyheteroaromatic structures in the O-CDs.

In general, pyridinium ylides are known to be versatile reagents in a broad range of reactions [55]. In particular, it is well documented that such ylides can give aromatic heterocyclic compounds in ring closure reactions with double-bond-containing dipolarophile molecules. Since such double-bond dipolarophiles can be formed as a product of the ring-opening reactions (Scheme 1, Path *b*), the formation of polyheteroaromatic structures in the core of the CDs can be explained by invoking reactions among deprotonated and ring-opened CPC molecules (Scheme 2). Moreover, analogous processes may involve not only CPC molecules, but also the formed 2-pyridone derivatives (e.g., the NH2P) that can be similarly deprotonated, and subsequently favor the ring-closure reaction of the dipolarophiles. Finally, the CDs obtained through such a mechanism can be also expected to be preferentially oil-dispersible. In fact, since the cyclization reactions above described affect only the close proximity of the head-groups of the CPC or the NH2P, the surface of the resulting CDs is expected to feature the alkyl chains that do not take part in the reactions, thus making the CDs much more suited to being dispersed in organic solvents less polar than water.

### 3.2. Synthesis of CDs Under Mild Heating

In principle, the described synthesis based on CPC reacting with NaOH does not require any external energy supply (e.g., heat, microwaves, ultrasounds, etc.) since the reaction can be activated just by the heat generated when the weakly acidic CPC solution (pH ≈ 5) is mixed with the concentrated NaOH solution (pH > 12). However, under these conditions, the kinetics of the O-CDs formation is rather slow, thus resulting in time-consuming synthesis (i.e., from tens of hours to days, depending on the concentration of NaOH in the reaction solution). Therefore, here, a mild external heat has been supplied to the system during the reaction process to possibly reduce the inherently long reaction time associated with the O-CDs synthesis run at RT. In particular, the NaOH addition has been carried out at a defined temperature (40 and 70 °C), and subsequently, the system has been kept at the same, constant temperature for the whole duration of the reaction.

**Figure 3:**
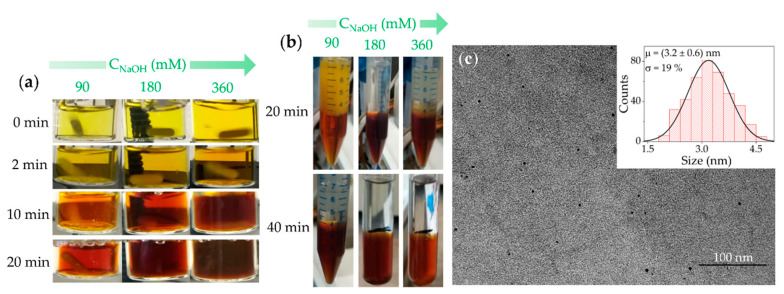
(**a**) Pictures of the vials containing the CPC initial solutions after the addition of 90, 180 and 360 mM NaOH solutions at 70 °C, at different reaction times; (**b**) Pictures of vials containing TPS after the separation procedure performed on the samples in (**a**) at reaction times of 20 and 40 min; (**c**) TEM micrograph (scale bar 100 nm) and size distribution analysis of the resulting CDs.

In the synthesis at RT, the obtained CDs display characteristic absorption bands in the visible range (Figure 1b), while bare CPC absorbs in the UV range at around 260 nm (Figure S1). Therefore, any observable color change in the reaction vessel is indicative of the CDs’ formation, and thus it can be used as a smart indicator to monitor the progress of the reaction [24]. Thus, variations in the reaction kinetics can be detected by a simple comparison of the color changes in the reaction mixture after the addition of NaOH at increasing concentrations. When performed at 40 °C, the reaction is found to proceed without any significant difference with respect to that run at RT (data not reported). Remarkably, a significant increase in the reaction kinetics becomes apparent when the synthesis is carried out at 70 °C. Indeed, upon the NaOH addition at 70 °C, the initially colorless CPC solution quickly turns yellow, irrespective of the C_NaOH_ (Figure 3a). Then, for reaction times above 10 min, the mixture goes brownish, getting darker and darker as the C_NaOH_ increases. Interestingly, from 10 min onwards, the mixture with the highest C_NaOH_ (360 mM) becomes turbid, thus suggesting, according to [24], the formation of insoluble O-CDs. When the synthesis is run at RT, the same result was reached after just 20 h.

Figure 3b shows the two-phase systems (TPS) obtained by quenching the reaction after 20 and 40 min, respectively, by adding immiscible CH_2_Cl_2_ in order to extract the O-CDs by centrifugation (Figure 3a). In such TPS, the O-CDs are dispersed in the bottom denser CH_2_Cl_2_ phase, thus remaining completely separated from possible residual W-CDs. The latter, if present, stay dispersed in the top aqueous phase. Thus, a visual inspection of the TPS will already provide a rough estimation of the extent of the conversion of W-CDs into O-CDs [24]. A rather complete conversion, with a brown lower organic phase and an almost colorless upper aqueous phase, is found to occur in just 20 min when 360 mM NaOH is added, and in 40 min in the presence of 90 and 180 mM NaOH (Figure 3b). The reaction run at RT takes remarkably longer to lead to a TPS with a W-CD-free, colorless aqueous phase. In fact, a minimum reaction time of 96 h was reported [24] with high C_NaOH_ (360 mM), while an even longer reaction time was needed for the lower C_NaOH_. Therefore, the proposed mild heating of the reaction mixture results in remarkably faster reaction kinetics. Finally, the TEM analysis shows that the average size of the obtained CDs is retained when synthesized at 70 °C, irrespective of the C_NaOH_ and of the reaction time (Figure 3c). Thus, the overall results demonstrate that when the synthesis is run at 70 °C, it is possible to shorten the reaction time from 96 h down to 20 min, while still preserving the average size of the O-CDs. 

### 3.3. Spectroscopic Characterization of CDs Synthesized at 70 °C

The absorption and emission properties of the O-CDs and W-CDs synthesized at 70 °C using different C_NaOH_ (90–360 mM) at increasing reaction times (from 15 to 60 min) have been investigated. As shown in Figure 3b, the W-CDs can be isolated within the first 20 min of reaction only when the carbonization process leading to the O-CDs occurs not too fast, that is, when a NaOH solution with a concentration in the range 90–180 mM is used. Otherwise, with higher C_NaOH_ or longer reaction times, only O-CDs are obtained. After the extraction of the organic phase, the O-CDs were purified following the procedure described above. Otherwise, dialysis was performed on the W-CDs collected in the aqueous phase so as to remove the possible blue-emitting intermediates and unreacted precursor. 

**Figure 4:**
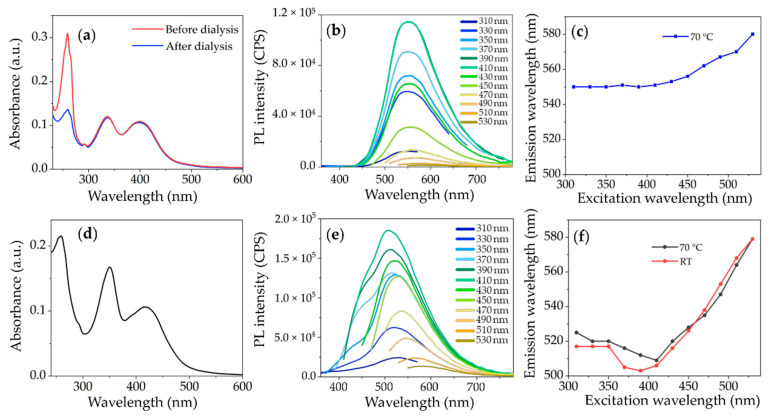
(**a,d**) UV-Vis absorption spectra of representative samples synthesized at 70 °C of W-CDs before and after purification (**a**) and O-CDs (the spectrum does not change after purification) (**d**); (**b,e**) PL spectra recorded with varying the λ_exc_ of the respective aqueous and organic phases after separation; (**c,f**) trend of the spectra position of the emission peak as a function of the λ_exc_ of W-CDs and O-CDs, respectively. In (**f**) the data at RT are also reported for comparison.

The UV-Vis absorption spectra of the W-CDs and O-CDs (Figure 4a,d) dispersed in water and CHCl_3_, respectively, after purification, are both characterized by an absorption peak below 300 nm and two bands in the visible region. The absorption spectrum of the W-CDs presents a peak at 260 nm that is significantly less intense, though still detectable, after the purification by dialysis, thus demonstrating the effectiveness of the purification process in the removal of most of the unreacted CPC. The residual weak absorption at 260 nm, observed in the purified sample, can be ascribed to π-π* transitions characteristic of the pyridinium rings present either in the carbogenic core of the CDs or in the residual unreacted CPC somehow interacting with the surface of the CDs that were not completely removed in the purification step. On the other hand, the characteristic absorption bands of the purified W-CDs, not affected by the dialysis, are centered at 337 and 400 nm, blue-shifted with respect to the correspondent signals in the O-CDs spectra, at 350 and at 420 nm, respectively. Such bands can be attributed to the surface chemical groups (e.g., carboxyl or pyridinium groups [23,24]) present at the surfaces of both types of CDs, in agreement with what was found at RT. In particular, the solvatochromic behavior of the bands, i.e., the hypsochromic shift observed for the CDs in water, allows us to unambiguously assign the signals to the n-π* transitions of the surface groups [29,42,44,45].

The PL spectra of the W-CDs (Figure 4b) do not change after their purification by dialysis (PL spectra of purified W-CDs are shown in Figure S3), exhibiting a single emission peak at around 550 nm that weakly red shifts for higher λ_exc_, and keeps its position when λ_exc_ is in the range 310–410 nm (Figure 4c). Such spectral positions of the PL bands of the W-CDs are also in agreement with those reported in [24] for the W-CDs synthesized in ambient conditions. The characteristic PL band of the O-CDs is peaked at 525 nm for λ_exc_ = 300 nm, and systematically blue shifts when λ_exc_ increases up to 410 nm. Conversely, a progressive red shift is observed for λ_exc_ > 410 nm. An analogous excitation-dependent behavior is also shown for the PL band of the O-CDs synthesized at RT, after extraction and purification (shown in Figure 4f for comparison). Hence, although the emission of the residual 2-pyridone derivatives, partially overlapping the green emission band of the O-CDs, may slightly alter the positions of the PL maxima, the emission of the O-CDs does not change significantly when the synthesis is run at 70 °C (Figure 4f).

Remarkably, no emission is detected around 400 nm, in the blue region of the W-CDs PL spectra, either before or after the purification, thus indicating that the blue-emitting fluorophores, even if present in the as-prepared samples, do not partition in the upper aqueous phase upon separation. Conversely, such blue emission is distinctively observed in the oil phase, as a small residue after the purification (Figure 4e). Such evidence supports the proposed reaction mechanism that assumes the 2-pyridone fluorophores acting as intermediates in the formation of the O-CDs, through reactions occurring in proximity to the pyridinium derivative head-group.

The different trend observed in the curves in Figure 4c, can also be partially attributed to the fact that intermediate fluorophores contribute only to the PL spectrum of O-CDs. The almost excitation-independent emission displayed by the W-CDs when exciting at 310–410 nm can be attributed to the surface-related states absorbing at around 335 nm, originating at the boundary between the carbogenic core and the functional groups (─COOH, ─C═O, ─OH, ─C═N) at the edge/surface of the CDs, having a lower energy than that of the carbogenic core levels [28]. Conversely, the progressive red-shift for λ_exc_ > 410 nm can be associated with the PL emission of surface-related states, originating from the broad absorption band centered at 400 nm, which involves lower surface energy state transitions in the surface-exposed functional groups that are also affected by different chemical environments. 

Since the surface functional groups of the W-CDs are also present in the O-CDs [24], the surface-/edge-related states are expected to determine the green emission of the O-CDs as well. However, for 310 nm < λ_exc_ < 410 nm, the heterogeneous and spectral overlapping contribution of the residual molecular fluorophores that are still present in the purified O-CDs modifies the excitation-dependent behavior of the PL spectrum, thus leading to the different trend observed in Figure 4f. Vice versa, the progressive red-shift of the PL peak observed for λ_exc_ > 410 nm can be also due to the formation of supramolecular aggregates of the molecular fluorophores emitting in the yellow region [28,29,56,57].

Finally, both the W-CDs and O-CDs exhibit characteristic emission properties and excitation-dependent behavior, and their absorption and emission features do not appear to be modified when the synthesis is run at 70 °C (Table S1). Therefore, the mild heating is capable of significantly speeding up the reaction, leaving unchanged the spectroscopic properties of the W-CDs and of the O-CDs. Such an approach is particularly suited to the fast preparation of the O-CDs, characterized by emission properties improved with respect to the W-CDs. Indeed, the measured absolute PLQY of the synthesized W-CDs is never higher than 2.3 ± 0.1%, while the O-CDs present a PLQY of 16 ± 1%, irrespective of the reaction conditions. The residual blue component (<10%) of the O-CDs emission has been evaluated by using a spectral deconvolution of the bands (e.g., Figure S2) to obtain a reliable PLQY value of the green component only. Such a PLQY value is comparable to that measured by Zheng et al. (16.7%) for the O-CDs synthesized at RT [24].

## 4. Conclusions

This work, investigating a facile and energy-saving synthetic approach for the preparation of CDs, based on CPC as the principal carbon precursor, contributes to explaining why such a precursor is so effective and convenient for the realization of CDs. The CDs are synthesized simply by adding NaOH to a CPC aqueous solution at RT or, alternatively, under mild heating in a thermostatic bath. Firstly, the study provides original insights into the chemical reactions responsible for the CDs formation, demonstrating the occurrence of intermediate blue-emitting molecular fluorophores. Moreover, the origin of such emitting species is explained on the basis of a sound formation mechanism, compatible with the chemistry of the precursor in the reaction environment and with the evidence reported in the literature. Indeed, 2-pyridone derivatives originate upon the oxidation or disproportionation of CPC in the presence of OH^−^ ions, finally evolving into the O-CDs. The investigation into the formation of the blue-emitting intermediates and their evolution over the reaction course has been essential in elucidating the CPC carbonization process, and in further understanding the overall recombination pathways that lead to the final emission characteristics. The proposed purification procedure has been demonstrated to be suitable for removing such blue-emitting species, and ultimately eliciting the actual O-CDs’ emission. 

Finally, a strong reduction in the inherently long reaction time (tens of hours to days) needed for the complete conversion of the W-CDs into O-CDs in ambient conditions has been accomplished. Indeed, the O-CDs have been demonstrated to be efficiently obtainable within 20 min, at the highest investigated C_NaOH_ (the higher the NaOH concentration, the faster the reaction), by simply keeping the reaction mixture at 70 °C, while their morphological and spectroscopic properties were preserved. Remarkably, such an improvement enables a fast synthesis of CDs, confirming the viability of such a unique synthetic strategy for producing CDs in a fast, reproducible, cost-effective and sustainable way. Finally, the dispersion in organic solvents allows the incorporation of the CDs into host polymers for nanocomposite preparation, and their fabrication as thin film [58,59] or further micromachining, thanks to the convenient film-forming properties of suitable organic solvents and their compatibility with clean room manufacturing. Such advanced processability paves the way for an effective integration of the luminescent CDs into photonic devices, LEDs or sensors [56,60,61].

## Supplementary Materials

The following are available online at https://www.mdpi.com/1996-1944/13/17/3716/s1, Details of synthetic and post-synthetic protocols, Figure S1: Absorption and PL spectra of a bare CPC solution, Figure S2: Deconvolution of a typical PL spectrum of purified O-CDs, Figure S3: PL spectra and peak positions as a function of λ_exc_, of W-CDs synthesized at 70 °C after purification; Table S1: Summarized spectroscopic data of the CD samples.
