# Supplementary material for: Oil-Dispersible Green-Emitting Carbon Dots: New Insights on a Facile and Efficient Synthesis
Source: Materials (Basel). 2020 Aug 22;13(17):3716. doi: 10.3390/ma13173716 (PMC7503313; doi:10.3390/ma13173716)
Supplement: Supplementary file 1 [file materials-13-03716-s001.pdf]

materials

Article

Oil-Dispersible Green-Emitting Carbon Dots: New Insights on a Facile and Efficient Synthesis

Gianluca Minervini [1,2], Annamaria Panniello [3,*], Elisabetta Fanizza [1,3], Angela Agostiano [1,3], Maria Lucia Curri [1,2,3,*] and Marinella Striccoli [3]

1 Chemistry Department, University of Bari "Aldo Moro", Via Orabona 4, 70126 Bari, Italy; g.minervini8@studenti.uniba.it (G.M.); elisabetta.fanizza@uniba.it (E.F.); angela.agostiano@uniba.it (A.A.)
2 Italian National Interuniversity Consortium of Materials Science and Technology (INSTM) Bari Unit, Chemistry Department, University of Bari "Aldo Moro", Via Orabona 4, 70126 Bari, Italy
3 CNR-IPCF-Bari Division, c/o Chemistry Department, University of Bari "Aldo Moro", Via Orabona 4, 70126 Bari, Italy; m.striccoli@ba.ipcf.cnr.it
* Correspondence: a.panniello@ba.ipcf.cnr.it (A.P.); marialucia.curri@uniba.it (M.L.C.)

Received: 20 July 2020; Accepted: 17 August 2020; Published: date

SUPPLEMENTARY MATERIALS

Synthetic and Post Synthetic Protocols

In a typical synthesis, a proper volume of a 2 M NaOH aqueous solution has been added to a fixed volume of a 20 mM CPC aqueous solution, thus to obtain final concentrations of precursor and NaOH of 15 and in the range 15–360 mM, respectively. All the reactions have been performed in common glass vials and air atmosphere. The ratio of NaOH to CPC has been changed by varying the volume of added NaOH solution while keeping fixed the total volume of the reaction batch (10 mL). The reaction has been carried out at room temperature (RT) or by pre-heating the CPC precursor solutions (at 40 and 70 °C) in a thermostatic bath before the NaOH addition and keeping the temperature constant for the whole duration of the reaction.

After the NaOH addition, the system has been left to react under magnetic stirring, until the reaction has been stopped, at the desired time. The quenching of the reaction was performed through pH neutralization, adding concentrated HCl aqueous solution (2 M). In general, at the end of the reaction both W-CDs and O-CDs have been obtained as products in the reaction batch.

The W-CDs and the O-CDs have been separated in a post synthetic procedure, by adding a fixed volume (10 mL) of apolar solvent ($CHCl_3$ or CH_2Cl_2) and centrifuging at 6000 rpm until obtaining a complete separation of the water and oil phases (20–40 min, depending on NaOH concentration and reaction time).

Subsequently, the extracted aqueous phase has been transferred into a semipermeable membrane (MWCO = 12,400 Da) and purified by dialysis overnight. A purification procedure was also required for the O-CDs (dispersed in $CHCl_3$ or CH_2Cl_2) in order to separate them from the 2-pyridone molecular fluorophores and other reaction intermediates. The procedure consists on double centrifugation cycle, each at 9000 rpm for 20 min at RT. Firstly, the raw O-CDs dispersion has been undergone under nitrogen flux to let the organic solvent ($CHCl_3$ or CH_2Cl_2) to evaporate. Then, to the residual pellet has been added a non-solvent, being it able to disperse the 2-pyridones and other reaction by-products, while resulting a poor solvent for the O-CDs. Thus, at the end of the centrifugation steps, the purified O-CDs have been recovered as a precipitate, and finally dispersed in the desired solvent (here, $CHCl_3$).

Spectroscopic Characterization of CPC Aqueous Solution

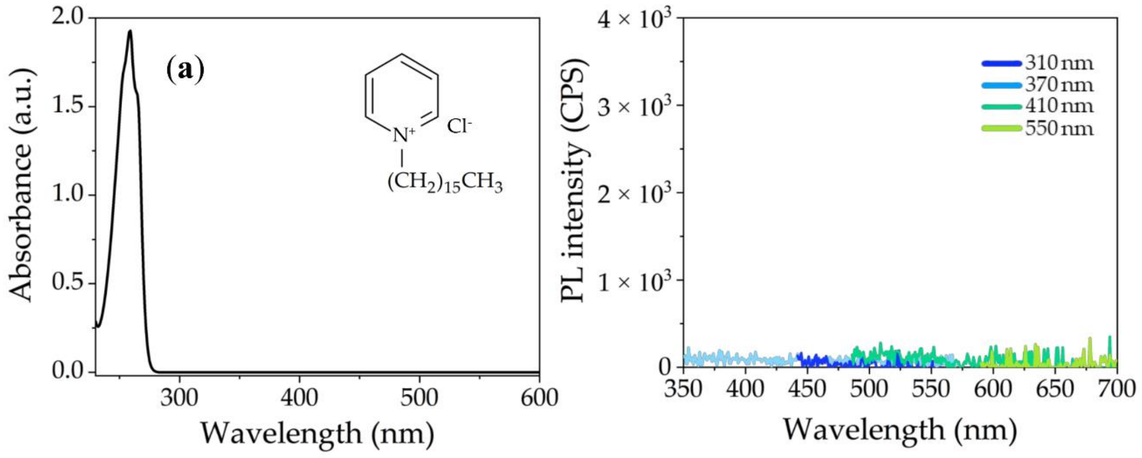

Figure S1. Absorption and PL spectra at different excitation wavelength of a bare CPC aqueous solution.

Deconvolution of Purified O-CDs PL Spectrum

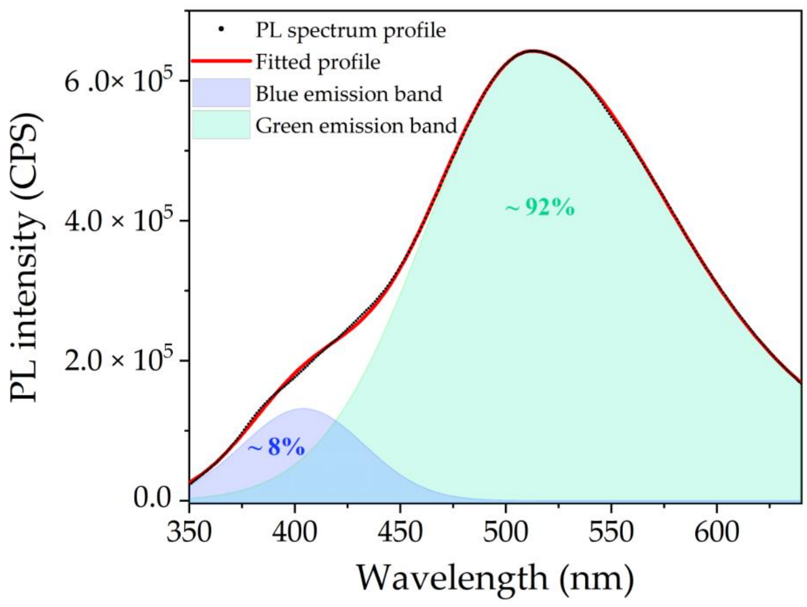

Figure S2. Deconvolution of a typical PL spectrum of purified O-CDs, pointing out the amount of residual 2-pyridone fluorophores. The whole emission profile has been fitted using Voigt functions to model the two PL bands in the blue and in the green region.

Emission Properties of W-CDs Synthesized at 70 °C

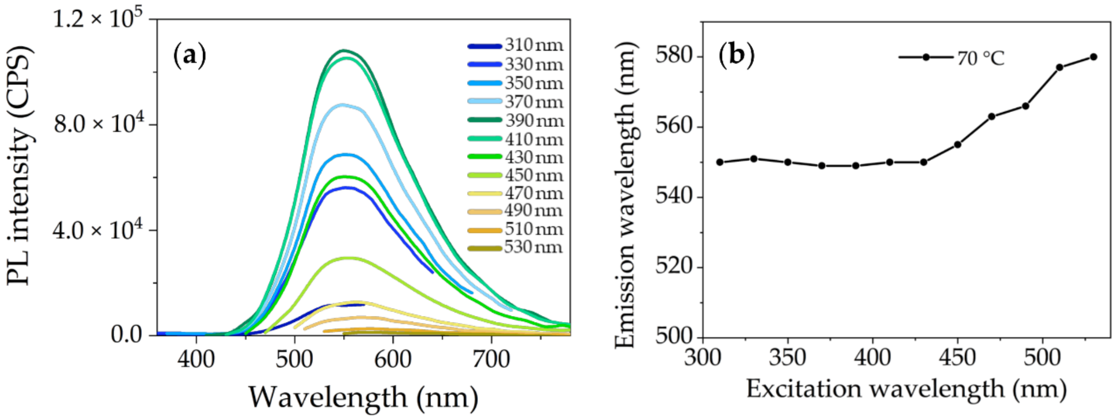

Figure S3. (**a**) PL spectra at different excitation wavelengths of the W-CDs collected in the synthesis at 70 °C after purification; (**b**) Trend of the emission peak as a function of the λ_{exc} for the PL spectra in (**a**).

Summary of CDs Spectroscopic Features

Table S1. Spectroscopic data of the CD samples.

Sample	UV-Vis Abs Peak Wavelength (nm)	PL Peak Position (Excitation Range) (nm)	Absolute PLQY
Reaction mixture (C_{NaOH} = 90 mM)	260, 360, 420	547 (410); 590 (500)	/
W-CDs; T = 70 °C	259, 337, 400	550 (310–410)<br>553 → 580[1] (430–530)	2.3 ± 0.1%
O-CDs; T = 70 °C	260, 350, 420	525 → 509[1] (310–410)<br>520 → 579[1] (430–530)	16 ± 1%
O-CDs; R.T.	260, 350, 420	517 → 506[1] (310–410)<br>516 → 579[1] (430–530)	16 ± 1%,

[1] peak position shifts in the indicated range at increasing λ_{exc}.

© 2020 by the authors. Submitted for possible open access publication under the terms and conditions of the Creative Commons Attribution (CC BY) license (http://creativecommons.org/licenses/by/4.0/).
